# Identification and Characterization of a Novel *Plasmodium falciparum* Merozoite Apical Protein Involved in Erythrocyte Binding and Invasion

**DOI:** 10.1371/journal.pone.0001732

**Published:** 2008-03-05

**Authors:** Thilan Wickramarachchi, Yengkhom S. Devi, Asif Mohmmed, Virander S. Chauhan

**Affiliations:** Malaria Group, International Centre for Genetic Engineering and Biotechnology, New Delhi, India; Queensland Institute of Medical Research, Australia

## Abstract

Proteins that coat *Plasmodium falciparum* merozoite surface and those secreted from its apical secretory organelles are considered promising candidates for the vaccine against malaria. In the present study, we have identified an asparagine rich parasite protein (PfAARP; Gene ID PFD1105w), that harbors a predicted signal sequence, a C-terminal transmembrane region and whose transcription and translation patterns are similar to some well characterized merozoite surface/apical proteins. PfAARP was localized to the apical end of the merozoites by GFP-targeting approach using an inducible, schizont-stage expression system, by immunofluorescence assays using anti-PfAARP antibodies. Immuno-electron microsopic studies showed that PfAARP is localized in the apical ends of the rhoptries in the merozoites. RBC binding assays with PfAARP expressed on COS cells surface showed that it binds to RBCs through its N-terminal region with a receptor on the RBC surface that is sensitive to trypsin and neuraminidase treatments. Sequencing of PfAARP from different *P. falciparum* strains as well as field isolates showed that the N-terminal region is highly conserved. Recombinant protein corresponding to the N-terminal region of PfAARP (PfAARP-N) was produced in its functional form in *E. coli*. PfAARP-N showed reactivity with immune sera from individuals residing in *P. falciparum* endemic area. The anti-PfAARP-N rabbit antibodies significantly inhibited parasite invasion in vitro. Our data on localization, functional assays and invasion inhibition, suggest a role of PfAARP in erythrocyte binding and invasion by the merozoite.

## Introduction

Malaria is still a major parasitic disease despite efforts spanning more than a century to control or eradicate it. Every year about 300–500 million people get infected with malaria causing about 1–2 million deaths [1]. Most of the clinical symptoms of *P. falciparum* malaria are attributed to the continuous cycles of asexual reproduction within the human erythrocytes that involve merozoite invasion, growth and schizogony. Merozoite invasion involves a series of highly specific, sequential interaction between merozoite and erythrocyte surface proteins, and is a crucial step in the parasite life cycle. Understanding the complex process of *P. falciparum* merozoite invasion requires identification and characterization of numerous potential parasite ligands and their interactions with receptors on RBC. These include different proteins on the surface of the merozoite that are possibly involved in weak initial attachment with the RBCs, as well as those protein that are released from the three apical secretory organelles of the merozoite, the rhoptries, micronemes and dense granules, prior to or during the host cell invasion and are involved in secondary interactions [2]. A number of these antigens are considered as promising vaccine candidates and some of these are presently at various stages of development for clinical trials [3]. However, it has been suggested that the most successful approach will require a combination of antigens involved at different stages of invasion. In addition, identification of new target antigens is also important for the development of future vaccines, since no fully protective vaccine has been assembled so far. Availability of *P. falciparum* genome sequence and proteome data has provided new opportunity to identify novel drug and vaccine target candidates. Recently, transcriptome analysis of the complete asexual intraerythrocytic developmental cycle (IDC) of *P. falciparum* identified 262 ORFs that showed sharp induction of expression during late schizont stages as in case of some of the well characterized merozoite surface/apical proteins that play role in merozoite invasion and are the best-known malaria vaccine candidates [4]. Of the 262 ORFs, 189 are of unknown function and represent a list of new putative vaccine candidate antigens. However it remains to be determined whether some of these proteins are localized on the merozoite surface/apical organelles and are involved in merozoite invasion process. Two main characteristics of the proteins localized on the surface or apical organelles are the presence of an N-terminal signal sequence and the presence of a C-terminal attachment motif such as GPI anchor or transmembrane region. Here, we have identified and characterized a novel merozoite protein that contains both the N-terminal signal sequence and a C-terminal transmembrane region. We have localized this protein in the apical region of the merozoite and named it as apical asparagine rich protein (PfAARP). We have also attempted to investigate the role of PfAARP during the erythrocyte invasion.

## Results

### Identification and sequence analysis of PFD1105w -an asparagine rich protein of *P. falciparum* (PfAARP) with C-terminal transmembrane region

**Figure 1:**
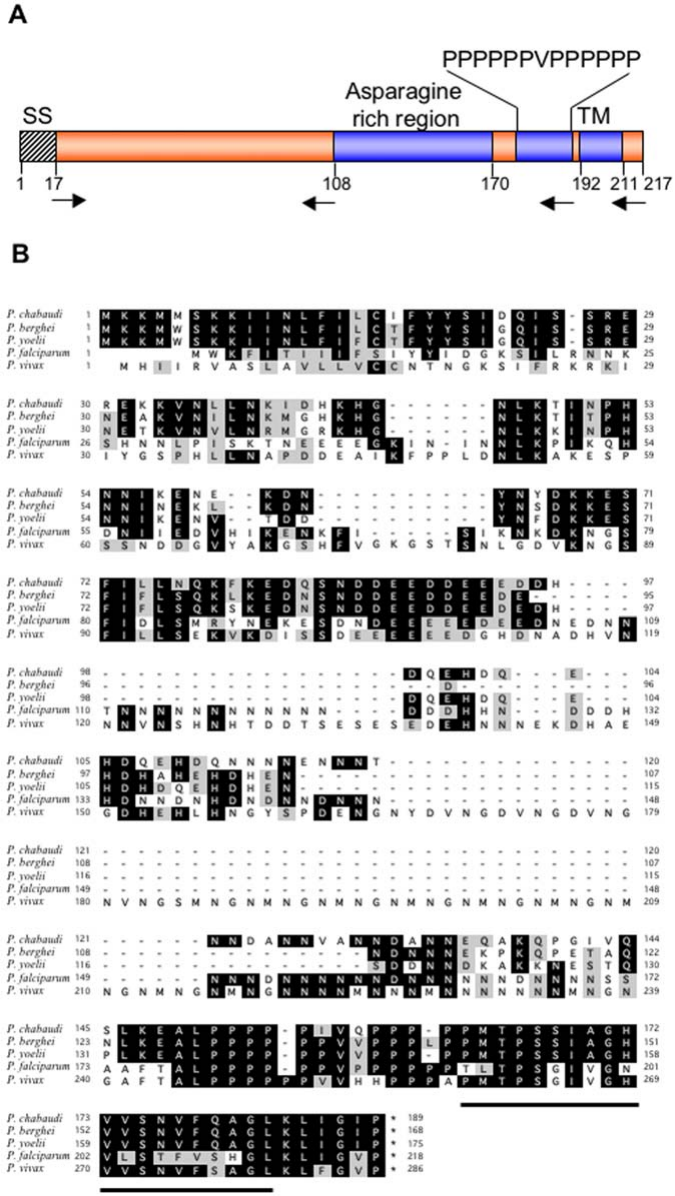
(A) Schematic representation of structure of PfAARP (Gene ID PFD1105w) gene showing location of signal sequence (SS) and trans-membrane (TM) region. The locations of asparagine rich region and conserved proline repeat region are also marked, respective amino acid positions are also indicated. (B) Amino acid sequence alignment of PfAARP with that of four homologs from *P. berghei* (PB402266.00.0) *P. chabaudi* (PC401501.00.0) *P. vivax* strain SaI-1 (Pv090210) and *P. yoelii yoelii* strain 17XNL (PY06454). Amino acids that are identical in at least three of five species (>60%) are shown in dark, amino acids that are similar in at least three of five species (>60%) or to those shown in dark, are shaded light grey. Transmembrane region is indicated by solid bar.

In our efforts to identify novel merozoite surface/apical organelle proteins that might be involved in merozoite attachment and invasion of RBC, we in silico screened *P. falciparum* proteome database. *P. falciparum* gene PFD1105w that codes for a hypothetical protein was selected as a candidate gene based upon its structural motifs and stage specific transcriptional profile that is similar to 28 other *P. falciparum* antigens which have been previously shown to play role in the process of merozoite invasion [4]. PFD1105w is a 217 amino acid long protein that contains a putative N-terminal hydrophobic signal sequence and a C-terminal transmembrane domain. Other interesting features include an unusually high asparagine rich region of 63 residues (108aa-170aa) in the C-terminal half of the protein and a run of proline residues (PPPPPPVPPPPPP) just before the transmembrane region (Figure 1A). Homologs of PFD1105w were identified from *P. berghei* (PB402266.00.0), *P. chabaudi* (PC401501.00.0), *P. vivax* strain SaI-1 (Pv090210) and *P. yoelii yoelii* strain 17XNL (PY06454) from the genome database. An alignment of the predicted proteins sequences of these genes is shown in Figure 1B. Common feature among these proteins are the presence of an N-terminal signal sequence and a stretch of proline residues near the C-terminus. *P. berghei*, *P. chabaudi* and *P. vivax* homolog also contain asparagine rich region, which varies in length from 6–18 residues. The homolog in *P. vivax* contains extra repeats of DVNG and GNMN residues just before the asparagine rich region.

### Expression of N-terminal fragment of PfAARP and specificity of antisera

An N-terminal fragment of PfAARP (PfAARP–N; 20aa-107aa) was cloned in pET28a vector and expressed in *E. coli*. The recombinant protein was purified to homogeneity that migrated as a single band on SDS-PAGE under reducing and non-reducing conditions (Figure S1A). RP-HPLC analysis of the protein on a C8 column revealed single symmetrical peak (Figure S1B). Polyclonal antibodies were raised in rabbits and mice against the recombinant protein. The specificity of the rabbit and mice antisera was assessed by Western blot analysis using parasite lysate from mixed stages parasite culture. The anti-PfAARP-N antibodies recognized a specific band (∼35 kDa) in the parasite lysate (Figure S2A). The molecular mass of PfAARP without the putative signal sequence is 22.4 kDa and its predicted pI is 4.3. The discrepancy in the predicted mass of the protein and band on the immunoblot is consistent with differences seen in other highly acidic proteins [5], [6]. Anti PfAARP antibodies did not react with the lysate of the uninfected RBCs. No band was detected in parasite lysate using pre-immune sera. (Figure S2B).

### Analyses of transcription and translation of PfAARP in asexual blood stage parasites

**Figure 2:**
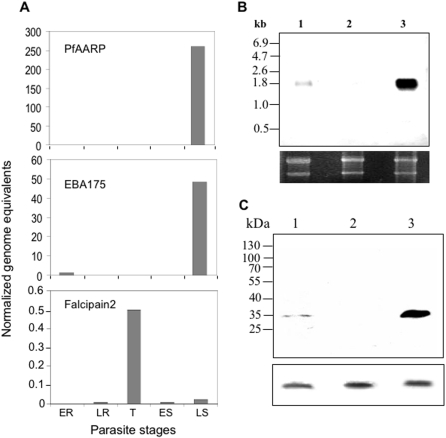
Stage specific expression of PfAARP in asexual blood stage parasites. (A) Relative transcription of PfAARP assessed by real-time-RT-PCR using total RNA extracted from tightly synchronized parasite cultures at early ring (ER), late ring (LR), trophozoite (T), early schizont (ES) and late schizont (LS) stages (8, 16, 30, 40 and 48 h after invasion). Stage specific expression of EBA-175 and Falcipain 2 was analyzed as controls. (B) Northern blot analysis of total RNAs isolated from synchronized parasite cultures at ring (lane 1), trophozoite (lane 2), and schizont (lane 3) stage hybridized with labeled PfAARP probe. Equal loading of RNA in all the wells was confirmed by ethidium bromide staining of rRNAs in the gels (lower panel). (C) Western blots analyses of equal number of highly synchronized parasites at ring (lane 1), trophozoite (lane 2) and schizont (lane 3) with anti-PfAARP antibodies. Anti-HRPII antibodies were used to probe a blot ran in parallel to show equal loading in each wells (lower panel).

To ascertain the expression pattern of PfAARP during asexual blood stage life cycle of the parasite, cDNAs were prepared from tightly synchronized parasite cultures at 8, 16, 30, 40 and 48 h after invasion and analyzed by real-time quantitative PCR using gene specific primers. As shown in Figure 2A maximum transcript level of PfAARP was observed in late schizont stages (48 h after invasion) whereas there was no detectable transcription in the early ring, late ring, trophozoites and early schizont stages (8, 16, 30 and 40 h after invasion respectively). Quantitative PCR from the same set of cDNA samples were also carried out for two other *P. falciparum* proteins, erythrocyte binding antigen-175 (EBA-175) and Falcipain 2 as controls. As expected EBA-175 transcript was also found to be maximum in cDNA samples from schizont stage parasites whereas Falcipain-2 showed maximum transcript level in trophozoite stage parasites. Northern blot analysis was also carried out using total RNAs from synchronized parasite cultures at ring, trophozoite and schizont stages, PfAARP DNA probe detected strong ∼1.8 kb message in the late schizont stage RNA (Figure 2B). A faint signal was also detected in ring stage RNA.

Western blot analysis using anti- PfAARP antibodies with total parasite lysates from cultures at different time point showed expression of PfAARP protein in late-schizont/merozoite stage parasites (Figure 2C), whereas it was not detected in parasites at trophozoite stages. In early ring stage parasite a faint band of PfAARP protein was observed that might represent left over proteins after invasion of the merozoites. Our results on transcription and translation analyses suggest that PfAARP is expressed in the blood stage parasites specifically in the late schizont/merozoite stages as in case of other merozoite integral membrane proteins.

### PfAARP localize to the apical end of the merozoites

**Figure 3:**
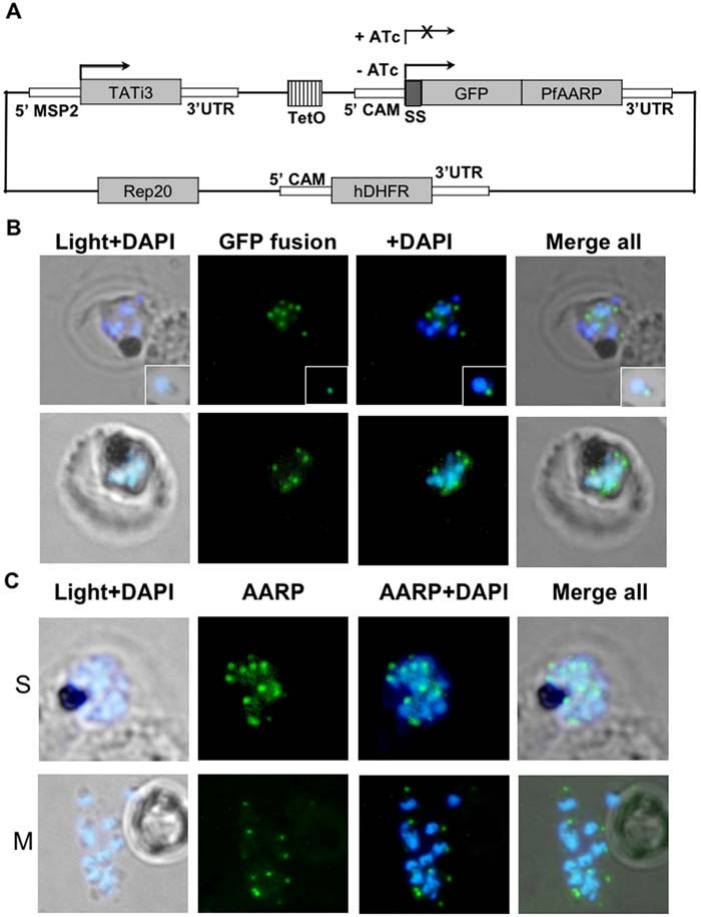
Localization of PfAARP to the apical end of the merozoites. (A) Schematic diagram of pTGFP-AARP plasmid construct containing selectable marker (human DHFR) under calmodulin promoter (5′ CAM), transactivator Tati-3 under MSP-2 promoter (5′ MSP-2) and chimeric gene consisting of secreted GFP (with signal sequence) and PfAARP under the control of Tet-responsive promoter. Expression of fusion gene is induced when ahydrotetracycline (ATc) is removed from the cultures. (B) Fluorescent microscopic images of transgenic parasites at schizont stages showing localization of PfAARP fused to a GFP reporter and expressed in an inducible system using schizont stage specific promoter. The parasite nuclei were stained with DAPI (blue). Enlarged images of selected individual free merozoite are shown in the insets. (C) Immuno-fluorescence assay to localize PfAARP in the schizont/merozoite stage parasites using anti-PfAARP (green) antibodies. The parasite nuclei were stained with DAPI (blue) and slides were visualized by fluorescence microscope. S, schizont and M, free merozoites.

To investigate the localization of PfAARP in the parasite, a GFP-targeting approach using an inducible expression system that directs strong, schizont-stage expression of transgene [7] was employed (Figure 3A). Expression of the fusion protein consisting of secreted GFP and PfAARP was activated in the transgenic parasites, by removing the repressor drug. These transgenic parasites were studied for localization of the PfAARP-GFP fusion protein. Fluorescence of the GFP-fusion protein was observed in a punctate manner in the schizonts, towards the apex of individual merozoites (Figure 3B). Immunofluorescence assays using PfAARP specific antisera also showed specific punctate staining in the schizont stage parasites (Figure 3C, panel I); in the free merozoites the staining was observed towards the apical ends (Figure 3C, panel III). No staining was observed in the ring and trophozoite stage parasites (not shown).

**Figure 4:**
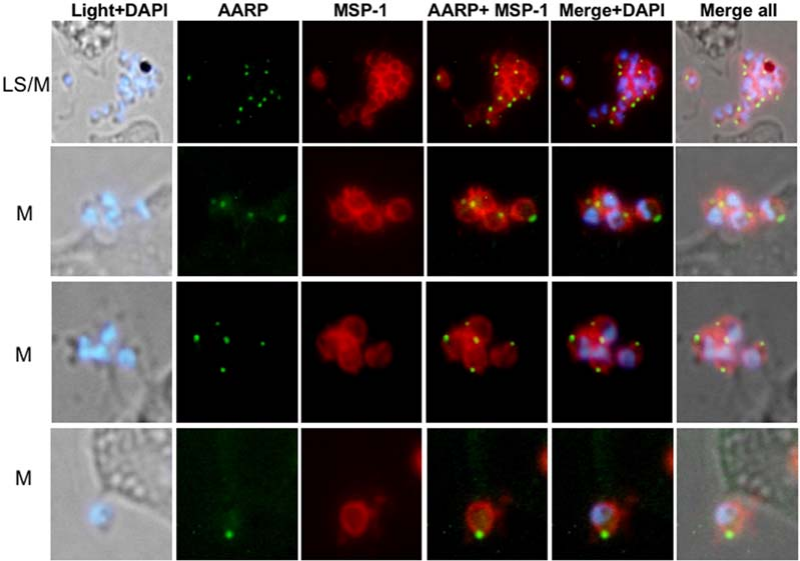
Immunofluorescence assay to localize PfAARP by coimmuno-staining of *P. falciparum* parasites with anti-PfAARP (green) and anti-MSP-1 (red) antibodies. The parasite nuclei were stained with DAPI (blue) and slides were visualized by fluorescence microscope. The apical ends of the merozoites have dense structure. MSP-1 staining was found around the merozoites and the PfAARP was localized at the apex of the merozoites. MS, mid schizont; LS, late schizont and M, free merozoites.

**Figure 5:**
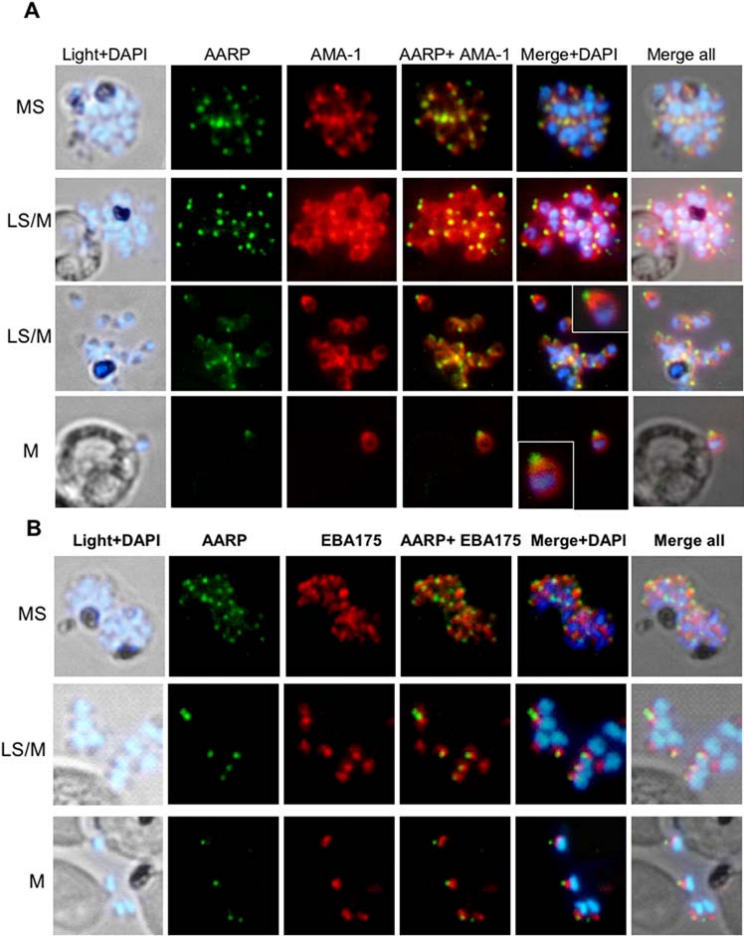
Spatial localization of PfAARP by co-immunostaining studies with microneme resident proteins AMA-1 (A) and EBA-175 (B). *P. falciparum* parasites were co-immunostained with anti-PfAARP (green) and anti-AMA-1 or anti-EBA-175 (red) antibodies. The parasite nuclei were stained with DAPI (blue). Both the microneme markers and PfAARP showed punctate staining in the schizonts. In the late schizonts and merozoites, AMA-1 was present over the entire surface of the merozoites but is most densely distributed at their apical tip, whereas EBA-175 staining was restricted to the apical ends. Enlarged image of selected individual merozoite is shown in the inset. MS, mid schizont; LS, late schizont and M, free merozoites.

**Figure 6:**
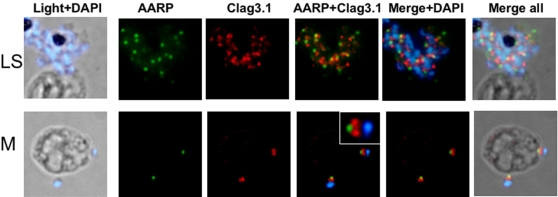
Spatial localization of PfAARP by co-immunostaining studies with rhoptry resident protein Clag3.1. *P. falciparum* parasites were coimmuno-stained with anti- PfAARP (green) and anti-Clag3.1 (red) antibodies. The parasite nuclei were stained with DAPI (blue). Clag3.1 staining was present in the rhoptry bulb in the free and invading merozoites (lower panel). PfAARP was localized just above these two rhoptry bulbs towards the apex of the merozoites. Enlarged image of merozoite invading in the host erythrocyte is shown in the inset. LS, late schizont and M, free merozoites.

**Figure 7:**
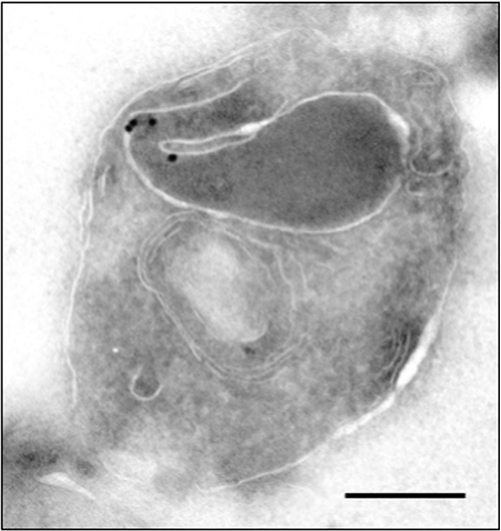
Localization of PfAARP by immuno-electron microscopy. Ultra thin sections of *P. falciparum* parasites at schizont/merozoite stages were labeled with anti-PfAARP antibody and gold labeled secondary antibody. Labeling was observed in the apical end of the rhoptries in merozoite. Scale bar = 250 nm.

To further define the localization of PfAARP in the merozoites, co-localization studies were carried out using antibodies against microneme [EBA-175 and apical membrane antigen-1 (AMA-1)] and rhoptry [cytoadherence-linked asexual gene (RhopH1/Clag3.1)] resident proteins as well as merozoite surface protein-1(MSP-1). Anti-MSP-1 antibody staining was found on the surface of merozoites with PfAARP staining localized at the tip of merozoites (Figure 4). AMA1 was present over the entire surface of merozoites but is most densely distributed at the apical tip (Figure 5A), whereas EBA175 tends to be restricted to the apical end (Figure 5B) as shown earlier [8], [9]. However, the PfAARP staining did not colocalize with EBA175, and showed only partial co-localization with AMA-1 (Figure 5A and 5B), suggesting that the PfAARP may not be present in the micronemes. The anti-Clag3.1 antibodies showed punctate staining in schizonts (Figure 6, panel I) and stained the two rhoptry bulbs in the free merozoites (Figure 6, panel II). The PfAARP staining did not colocalize with Clag3.1 staining in the schizont (Figure 6, panel I). In the invading merozoites, PfAARP staining was observed just above the two rhoptry bulbs, at the apical end facing towards the RBC membrane (Figure 6, panel II).These results suggest that PfAARP is present at the apical end of the merozoites close to the rhoptry neck. To confirm these results we carried out immuno-elctron microscopic studies using anti-PfAARP antibodies. PfAARP staining was found to be localized in the apical ends of rhoptries, close to the rhptry neck area, where the two rhoptries join in a common ductule (Figure 7). No staining was found in the rhoptry bulbs or any other organelle of the merozoites.

### PfAARP binds with human RBCs through its N-terminal region

The full length PfAARP gene and its N-terminal half that lacks the asparagine rich region were expressed on the surface of COS cells in order to test their binding with the human erythrocytes. The secretory signal sequence and transmembrane segment of *Herpes simplex* virus glycoprotein D (HSV gD) gene, in the pRE4 mammalian expression vector used [10], target these protein to the surface of transfected COS cells. A construct pHVDR22, designed to express *P. vivax* Duffy binding protein region II (PvRII) in same way [11], was used as the positive control in these assays. Immunofluorescence assay of the transfected cells using DL6 antibodies (see materials and methods) confirmed expression of these proteins on their surfaces. The full length PfAARP gene (construct pRE4-PfAARP-F) showed binding with human erythrocytes although it's binding efficiency were lower than that of PvRII construct (Table 1). The N-terminal half of PfAARP (construct pRE4-PfAARP-N) also showed RBC binding with equal efficiency to that of full gene (Table 1) suggesting that the RBC binding domain is present in the N-terminal half of PfAARP. Treatment of RBCs with chymotrypsin did not affect binding with the PfAARP expressing COS cells, whereas trypsin and neuraminidase treatment significantly reduced the binding. These results suggest that PfAARP binds with a receptor on RBC that is resistant to chymotrypsin treatment but is sensitive to trypsin and neuraminidase.

**Table 1:** Binding of normal and treated erythrocytes with Cos-7 cells expressing full length PfAARP or its N-terminal fragment, PfAARP-N

Construct	No. of rosettes^1^			
	Untreated	Trypsin	Neuraminidase	Chymotrypsin
PfAARP	52 (±8)	11 (±5)	16 (±4)	50 (±10)
PfAARP-N	54 (±3)	8 (±6)	14 (±6)	52 (±8)
pHVDR22 2	70 (±9)	N/A	N/A	5 (±2)

1 The number of COS-7 cells with rosettes of bound erythrocytes was scored in 20 fields at ×200 magnification. Number of rosettes of each experiment was normalized according to respective transfection efficiency to 5%. Mean value of 3 independent experiments is reported with standard deviation.

2 Construct designed to express *P. vivax* Duffy binding protein region II (PvRII) [11]

### Reactivity of PfAARP with human immune sera

**Figure 8:**
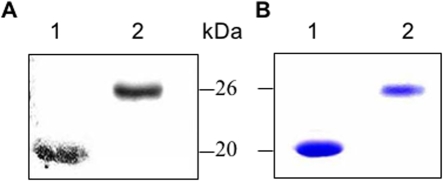
(A) Anti-PfAARP antibodies are present in human immune sera from *P. falciparum* endemic area. Western blot analysis showing reactivity of recombinant PfAARP-N (lane 1) with human immune sera, PfMSP-1_19_ (lane 2) was kept as a positive control. (B) Coomassie blue stain SDS- poly acryl amide gel ran in parallel.

In order to examine whether antibodies against PfAARP were elicited during natural infection with *P. falciparum*, recombinant PfAARP was examined for its reactivity with sera collected from individuals residing in *P. falciparum* endemic areas. Pooled sera from these individuals showed reactivity with the recombinant protein on a western blot (Figure 8). In ELISA, the recombinant protein was recognized by >50% of human sera samples that were also positive for reactivity with recombinant PfMSP-1_19_ protein, another leading blood stage malaria vaccine candidate antigen (Figure S4). These reactions were specific as no reactivity was observed in western blot as well as in ELISA with sera from individuals who had never been exposed to malaria.

### Conservation of gene encoding PfAARP in different *P. falciparum* isolates

To assess the level of conservation of PfAARP gene sequence in *P. falciparum*, PfAARP gene from five different *P. falciparum* strains and five field isolates were amplified. Sequencing of these genes showed that the N-terminal region of PfAARP is highly conserved among these isolates and only one residue at position 103 showed variation from N→S in four sequences; variation in the length of asparagine repeat region was observed among these isolates that varied from 51-63aa (25–28% of the total amino acid composition) (Figure S5). In addition, the transmembrane region and the proline stretch was also found to be highly conserved.

### Recombinant PfAARP-N binds with RBCs and anti- PfAARP-N antibodies inhibits this binding as well as erythrocyte invasion by the merozoites

**Figure 9:**
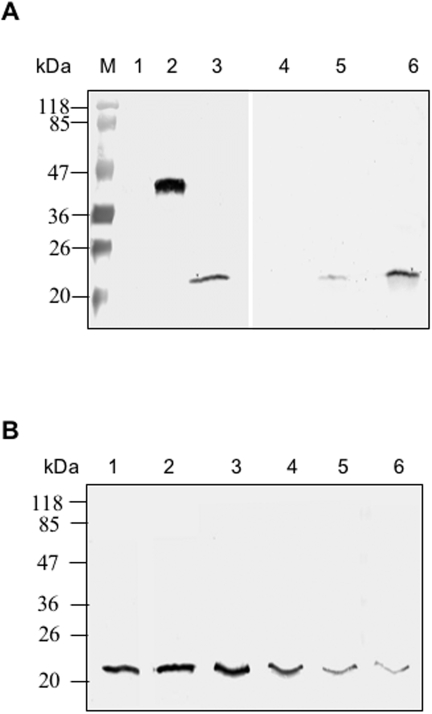
Erythrocyte binding assay using recombinant PfAARP-N and binding inhibition by anti-PfAARP-N antibodies. (A) Western blot using monoclonal anti penta histidine antibodies showing detection of recombinant proteins in elutes from the RBC binding assays of protein PfDH60 (negative control; lane 1), PvRII (lane 2) and PfAARP-N (lane 3) using untreated human RBC, and in elutes from similar binding assays of PfAARP-N using human RBCs treated with trypsin (lane 4), neurminidase (lane 5) and chymotrypsin (lane 6). (B) Western blot of elutes from the RBC binding assays with the recombinant PfAARP-N protein pre-incubated in RPMI alone (lane 1) or with antibodies purified from pre-immune sera (100 µg, lane 2) and anti- PfAARP antibodies purified from rabbit immune sera (10, 25, 50 and 100 µg, lane 3–6). Equal amount of recombinant protein was used in each assay.

As the RBC binding domain of PfAARP was found to be present in its N-terminal half, the recombinant protein corresponding to this region, PfAARP-N, was tested for its ability to bind with the human erythrocytes in an in vitro assay to ascertain its functionality. Purified PfAARP-N and *P. vivax* Duffy binding antigen region II (PvRII) bound with human erythrocytes in this assay whereas *P. falciparum* DNA helicase 60 (PfD60) did not (Figure 9A), validating that the recombinant PfAARP-N is functionally active. In addition, binding of recombinant PfAARP to human erythrocytes was sensitive to trypsin and neuraminidase treatments, as in case of COS cell surface binding assays (Figure 9A). Further, ability of the anti- PfAARP-N antibodies to inhibit binding of PfAARP-N with RBCs was assessed. As shown in Figure 9B, antibodies purified from rabbit immunized with PfAARP-N inhibited RBC binding of PfAARP-N in a dose dependent manner. No inhibition was observed with IgG purified from pre-immune sera.

The ability of anti-PfAARP-N antibodies to inhibit parasite invasion was assessed by an *in vitro* parasite growth inhibition assay. Anti-PfMSP-1_42_ antibodies were used as positive control. As shown in Table 2, anti-PfAARP-N antibodies significantly (up to 70%) inhibited the parasite growth in a concentration dependent manner. The invasion inhibition by anti-PfAARP-N antibodies was comparable to the inhibition by anti-PfMSP-1_42_ antibodies.

**Table 2:** Inhibition of *P. falciparum* invasion of erythrocytes by purified rabbit anti-PfAARP antibodies. Anti-PfMSP-1_42_ rabbit antibodies were used as a control.

Group	% of parasitaemia^1^				% of invasion inhibition^4^			
	Concentration of IgG (mg/ml)^2^				Concentration of IgG (mg/ml)			
	0.5	0.25	0.125	0.0625	0.5	0.25	0.125	0.0625
Adjuvant control	5.66 (±0.12)^3^	5.76 (±1.25)	6.13 (±0.32)	5.34 (±0.58)	-	-	-	
PfAARP immune	1.73 (±0.31)	2.63 (±0.58)	3 (±0.7)	3.24 (±013)	69.41	54.34	51.10	39.4
MSP-1_42_ immune	2.07 (±0.12)	1.93 (±0.12)	2.86 (±0.31)	3.4 (±0.01)	63.50	66.50	53.30	36.3

1 Parasitaemia is the average of triplicate wells.

2 Final concentration of IgG in a well

3 Standard deviation

4 Calculated against wells with antibodies from adjuvant control (chi-squares test, P≤0.01).

## Discussion

Availability of the predicted proteome and transcriptome data for *P. falciparum* has provided an impetus to find novel drug targets and vaccine candidate antigens. In the present study we have identified PfAARP as one such candidate antigen. Transcriptome analysis of stage specific asexual blood stage parasites grouped PfAARP with 28 other *P. falciparum* proteins that are involved in the process of merozoite invasion [4]. We have confirmed expression of PfAARP specifically at late schizont/merozoite stage by quantitative real-time PCR and western blot analysis, suggesting it to be essentially a merozoite protein. PfAARP contains an N-terminal signal peptide and a C-terminal transmembrane region. The N-terminal signal sequence in different *P. falciprum* proteins is responsible for entry of the proteins into the ER-trans Golgi network (TGN) secretory system and then these proteins get further distributed in to different trafficking routes depending upon additional signals [12], [13], [14], [15]. A number of merozoite proteins that are localized on its surface or reside in the apical secretory compartments, harbor N-terminal signal sequences. These protein after passing through the secretory pathway, get targeted to their final destination such as merozoite surface, rhoptries or micronemes [12], [13], [16]. Many of these merozoite surface proteins (MSPs) such as MSP-1, -2, -4, and -5 attach to the plasma membrane via a C-terminal glycosylphosphatidyl inositol (GPI) whereas all known micronemal proteins are type I integral membrane proteins that contain a C-terminal transmembrane domain and a short cytoplasmic domain [2]. The rhoptries also contain several resident proteins that either have a transmembrane region, such as rhoptry-associated proteins 1 (RAP1) [17] or have a GPI anchor such as rhoptry associated membrane antigen (RAMA) [18].

The structural features of PfAARP i.e. presence of a C-terminal transmembrane region in addition to the N-terminal hydrophobic signal sequence and its stage specific expression pattern, led us to speculate that it might be a putative merozoite surface/apical protein. To confirm this hypothesis, we studied localization of PfAARP by GFP targeting approach in a transgenic parasite line. A number of studies have used this approach to explore the localization and trafficking of the parasite proteins [14], [19], [20], [21]. However, correct timing of expression of the transgene in *P. falciparum* transgenic parasite lines has been shown to be a prerequisite for protein sorting and correct subcellular localization [12], [22]. In addition, constitutive over expression of some transgenes encoding merozoite surface proteins might be toxic to the parasite [7]. Therefore, to localize PfAARP in the parasite, a secreted GFP- PfAARP fusion protein was expressed in the parasite under the control of a schizont stage promoter of MSP-2 and a tetracycline-regulated transactivator, using a recently developed transfection vector [7], [23]. The temporal expression of the transgene in this system mimics that of MSP-2 [7] which has a similar transcriptional profile to PfAARP. The GFP- PfAARP fusion protein expressed in the transgenic parasite using this approach was found to be localized towards the apical end of the merozoites. Immunofluorescence assays and colocalization studies further confirmed presence of PfAARP at the apical end of the merozoites. Merozoites harbour a secretory complex at their apical end that consists of organelles like rhoptries and micronemes. These organelles contain many of the key proteins needed for directional attachment and invasion of the merozoite [2], [24]. After the release of merozoites from schizont and during the invasion process, contents of both rhoptries and micronemes get excreted through the ductules at the neck of the rhoptry [25], [26]. Rhoptries contain a number of proteins including high molecular mass proteins complex, RhopH complex, membrane associated Rhoptry proteins (RAMA) and rhoptry associated proteins (RAP1, RAP2 and RAP3) that are focus of interest as vaccine candidates. RhopH complex proteins localize to the basal bulb of the rhoptries [27] and are involved in erythrocyte binding and in establishment of parasitophorous vacuole [28]. RAMA has been shown to be localized to inner face of the rhoptry bulb membrane in close apposition of RhopH3 and RAP1 [18]. Micronemes contain several proteins involved in the process of merozoite invasion including duffy binding like proteins, EBA-175, EBA-140 and EBA-180, that binds with the host RBC in a sialic acid dependent manner [2]. In the present study, PfAARP did not colocalize with any of the micronemal and rhoptry proteins tested, and was found to be discretely localized above these apical secretory structures in the free merozoites. Our results of immuno-electron microscopic studies clearly show that PfAARP is situated in the apical ends of the secretory organelle rhoptries in the merozoites. Some other *P. falciparum* proteins have been localized to the rhoptry neck that may play role in binding and invasion of the merozoites. These include rhoptry neck protein PfRON4 that is a homologue of *Toxoplasma gondii* rhoptry neck protein TgRON4. PfRON4 forms a complex with PfAMA1 during its secretion in the course of merozoite invasion [29]. TgRON4 also interacts directly with the TgAMA1 and participate in the formation of moving junction, a circumferential zone which forms at the apical tip of the parasite during its invasion in the host cell [30]. *P. falciparum* also expresses a family of proteins (PfRh1, PfRh2a, PfRh2b, and PfRh4) that are orthologs of *P. vivax* reticulocyte binding proteins, these proteins also localize in the rhoptry neck at the merozoite apical end [2], [31], [32]. Recently, a GPI anchored merozoite protein Pf34 was also found to be localized to the rhoptry neck [33].

Since a number of merozoite apical proteins play role in binding and invasion of RBC by the merozoites [2], we tried to assess a possible role of PfAARP in RBC binding. Our results of binding assays with COS cell surface expressed PfAARP suggest that PfAARP is involved in binding of the merozoite with the host RBC. A number of surface proteins are expected to be involved in initial binding of the merozoite with the RBC whereas proteins of apical organelles such as the members of DBL family and homologs of reticulocyte binding proteins are involved in binding after re-orientation of the merozoite [2], [24]. The parasite utilizes a number of receptors on the erythrocyte surface that interact with these ligands including glycophorin A, B and C and unknown receptors E, X, Y and Z. These receptor–ligand interactions between the merozoite surface/apical proteins and erythrocyte surface receptors are characterized by their enzyme sensitivities to neuraminidase, trypsin, and chymotrypsin [31], [34]. We found that PfAARP binds with RBC in a chymotrypsin resistant manner, and shows sensitivity to trypsin and neuraminidase treatments suggesting that it utilizes a receptor similar to Glycophorin A or C on the RBC surface. Taken together the results of localization and binding assays suggested that the PfAARP is involved in binding of the merozoite after re-orientation during the process of invasion. Further the binding domain was localized in the N-terminal half, suggesting that the repeat structure in the C-terminal half has no role in RBC binding. Repeat regions are common in *Plasmodium* proteins located on the merozoite surface or in the apical secretory organelles [18], [31], [35], [36]. Although the role of these repeat regions is not clear, it has been suggested that these might act as immunodominant epitopes that may contribute to malaria immunity [37].

Malaria proteins show extensive polymorphism that is suggested to be one of the main strategies of the parasites to evade host immune mechanisms, and the antigens that are under natural immune pressure tend to have higher levels of polymorphism. [38], [39]. On the other hand, it is also shown that residues that show polymorphism due to immune pressure are different than the functionally critical domains which are relatively conserved and are more suitable target for vaccine development [40], [41]. Having found out that PfAARP is involved in RBC binding, we assessed if it is also conserved across different *P. falciparum* strains and field isolates. Sequencing of PfAARP gene from different *P. falciparum* strains and field isolates showed that it has evolutionary conserved N-terminal half that may signify the functional importance of the N-terminal region of PfAARP for the parasite survival. These results in combination with our RBC binding assays suggest that PfAARP plays a significant role in binding and invasion of RBC by the merozoite.

A number of merozoite surface antigens are proposed to elicit protection against the parasite by antibody mediated inhibition of merozoite invasion [42]. We assessed if there is any immune response generated against PfAARP during natural exposure to *P. falciparum*. The asparagine repeat region present in PfAARP is known to be common among many parasite proteins [43], [44], [45]. Therefore to avoid detection of cross reactive antibodies against these regions, we used recombinant protein corresponding to N-terminal half of the gene, PfAARP-N, that contains the functional RBC binding domain. Recombinant PfAARP-N was recognized by immune sera from individuals residing in *P. falciparum* endemic areas, indicating that it contains epitope(s) that are target of antibody response generated during natural exposure to *P. falciparum*. Presence of anti-PfAARP antibodies in human immune sera and conservation PfAARP gene across different isolates/strain suggest that this antigen may induce effective host-immune response against the parasite. We further evaluated the efficacy of rabbit antibodies generated against the PfAARP-N to inhibit merozoite invasion. The recombinant PfAARP-N was functional with respect to its binding activity with human erythrocytes and this binding was inhibited by anti- PfAARP-N antibodies in a dose dependent manner. These results suggested that binding inhibitory antibodies are present in the rabbit immune sera that block receptor binding function of PfAARP. These antibodies also significantly inhibited invasion of the erythrocytes by parasites in a dose dependent manner. Our invasion inhibition data using binding inhibitory anti- PfAARP antibodies suggest that binding of PfAARP to the RBC is a vital step during the process of merozoite invasion and support PfAARP to be a putative vaccine/drug target candidate. Owing to the complexity of the invasion process, it is essential that strategies for preventing malaria disease that are directed against merozoite invasion should include such parasite proteins that play important roles during different steps of invasion using diverse ligand-receptor interactions.

## Material and Methods

### Parasite culture, transfection plasmid construct and parasite transfection

*Plasmodium falciparum* strains 3D7 were cultured on human erythrocytes (4% hematocrit) in RPMI1640 media (Invitrogen) supplemented with 10% O+ human serum using standard protocol [46]. Parasite cultures were synchronized by two sorbitol treatments at 4 h apart following Lambros and Vanderberg [47]. To generate a transfection vector construct, the PfAARP gene was amplified from *P. falciparum* 3D7 genomic DNA using primers: 500A- 5′ CCG ACG CGT ATA TTG AGG AAT AAC AAA AGT CAT AAC 3′ and 501A- 5′ CGG ACT AGT TTA GGG TAC TCC GAT TAA TTT TAA ACC 3′. Amplified PCR product was digested with *Mlu*I and *Spe*I restriction enzymes and cloned in frame to the C-terminus of secreted GFP in the *Mlu*I and *Spe*I sites of the transfection vector [5] to give pTGFP-AARP transfection vector. This vector contains fusion protein of secreted GFP and PfAARP under tetracycline-inducible expression system. Synchronized *P. falciparum* 3D7 ring stage parasites were transfected with 100 µg of purified plasmid DNA (Plasmid Maxi Kit, Qiagen, Valencia, CA) by electroporation (310V, 950 µF) [48] and the transfected parasites were selected over 2.5 nM of WR99210 drug in presence of 5 mM Anhydrotetracycline.

### Isolation of DNA, total RNA, real time quantitative PCR, northern blot analysis

The genomic DNA was isolated from in vitro culture of *P. falciparum* following a standard protocol [49]. Total RNAs were isolated from synchronized *P. falciparum* 3D7 parasite cultures using mini RNA isolation kit (Qiagen). An aliquot of 50 ng of total RNA was used to synthesize cDNA using cDNA synthesis kit (Invitrogen) following manufacturer's recommendations. Gene specific primers were designed using Beacon Designer4.0 software, for the genes PfAARP (535A, 5′ AAc GAA TGA AGA AGA GGA AGG 3′ and 536A, 5′TCT CAT ACT TAA ATC AAT AAA GGA ACC 3′), EBA175 (EBA175RTF: 5′ AAT TTC TGT AAA ATA TTG TGA CCA TAT G 3′ and EBA175RTR: 5′GAT ACT GCA CAA CAC AGA TTT CTT G 3′) and Falcipain 2 (Fal2F 5′-GCTTGTAGGTTTT GGTATGAAAGAA-3′ and Fal2 R 5′ AGATAGGTCCCTTTTTAAAATACTATTGAC-3′) [50]; 18S rRNA control primers (18SF 5′-GCTGACTACGTCCCTGCCC-3′; 18SR 5′-ACAATTCATCATATCTTTCAATCGGTA-3′) were used following Blair et al. [51]. Quantitative real time PCR were carried out in triplicate using the iCycler version 3.0

(Bio-Rad); each reaction was containing equal amount of cDNA, 100 ng of both the gene specific primers and 1× SYBR Green PCR mix (Bio-Rad). Threshold cycle (Ct) values were calculated by using iCycler software. Standard curves for each gene were obtained by using different dilutions of wild-type gDNA (100–1 ng) as template, and these standard curves were used to determine genome equivalents of Ct values for every gene and 18S rRNA in each RNA sample [51]. Genome equivalents of each gene were normalized using that of 18S rRNA for all the RNA samples.

### Expression plasmid construct, expression and purification of recombinant protein, RP-HPLC and Generation of polyclonal anti-sera

An N-terminal fragment of PfAARP gene (20aa- 107aa) was amplified by PCR from *P. falciparum* 3D7 genomic DNA using primers 513A (5′ GGC *GGA TCC* ATA TTG AGG AAT AAC AAA AGT CAT 3′) and 514A (5′ GAC *AAG CTT* ATC TTC ATT GTC TTC TTC ATC 3′). The amplified fragment was digested with restriction enzymes *Bam*HI and *Hind*IIII and cloned in the *Bam*HI and *Hind*III sites of pET28a expression vector (Novagen). The resultant plasmid pET28a-PfAARP-N was transformed into expression cells BLR(DE3) for expression of the recombinant protein. These *E. coli* BLR(DE3) cells were grown in Luria broth containing tetracycline (25 µg/ml) and kanamycin (100 µg/ml) at 37°C with shaking to an OD_600_ of 0.6–0.7 and expression of recombinant protein was induced with isopropyl-β-thioglactopyranoside (IPTG) at a final concentration of 1 mM. The cultures were further grown at 37°C for 3–4 h and the *E. coli* cells were harvested by centrifugation. The cell pellet was suspended in lysis buffer (20 mM Tris pH 8.0, 500 mM NaCl, 1 mM benzamidine hydrochloride and 1% Tween 20) and the bacterial cells were lysed by sonication (Torebeo Ultrasonic Processor 36800, Cole Parmer). The lysate was centrifuged at 15,000×g for 30 min at 4°C and the supernatant was incubated with Ni-nitrilotriacetic acid (Ni^2+^-NTA) agarose resin (Qiagne), pre-equilibrated with the lysis buffer, at 4°C for 1 hr. The suspension was applied to a column and washed with 10 bed volumes of the wash buffer (20 mM Tris-HCl, pH 8.0, 250 mM NaCl 5 mM imidazole). The bound protein was eluted with 15 ml of elution buffer (20 mM Tris and 250 mM NaCl) containing 50 mM imidazole. The eluates were analyzed on SDS-PAGE and the fractions containing the recombinant protein with a clear single band were pooled and the protein concentration was determined using the BioRad protein assay system (BCA method) and a standard curve of bovine serum albumin. The purified protein was analyzed by reverse phase HPLC on C8 column using a linear gradient of 10 to 90% acetonitrile in water containing 0.05% trifluoroacetic acid.

To generate polyclonal anti-sera against PfAARP, female BALB/c mice were immunized (on day 0) with the purified recombinant protein (25 µg) formulated in complete Freund's adjuvant (Sigma, USA). The mice were administered two booster doses (day 14 and 28) of the proteins formulated in Freund's incomplete adjuvant. The mice serum was collected 10 days after the second boost. Two New Zealand white rabbits (3 months old) were immunized in the same way with 250 µg of recombinant protein (on day 0) and administered two booster doses (on day 28 and 49). The rabbit sera were collected on day56.

### ELISA and Western blot analyses

Antibody response in mice, rabbits and human serum samples were quantified by ELISA. Briefly, wells of flat bottom microtitre plates (Dynatech) were coated with 100 ng of the recombinant protein in 0.06 M carbonate-bicarbonate buffer (pH 9.6). The plates were washed thrice with 0.05% tween in PBS (PBS-T) for 5 minutes each and blocked with 5% skimmed milk in PBS for 1 h at room temperature. The antigen-coated wells were sequentially incubated with the appropriate dilutions of respective test sera and optimally diluted mouse anti-human-IgG horse radish peroxidase (HRP) labeled secondary antibody. The enzyme reaction was developed with o-phenylenediamine as a chromogen and hydrogen peroxide as a substrate prepared in citrate phosphate buffer, pH5.0. The reaction was stopped with 8N H_2_SO_4_ and OD_490_ was measured using an ELISA micro-plate reader (Molecular devices). Serial dilution of mice and rabbit sera samples were analyzed in triplicate by ELISA and the last dilution, at which the OD_490nm_ was greater than the mean OD_490nm_ plus two standard deviations from the pre-immunization serum were defined as the endpoint titer.

IgG specific for PfAARP in immune human sera samples were also assayed by ELISA using recombinant protein PfAARP-N. Each test serum sample was assayed at a dilution of 1∶20 against the proteins in duplicate with normal human sera as control as previously described [52]. The positive cut-off value was calculated as the mean OD value of the normal controls plus two standard deviations. Same battery of sera samples were used at a dilution of 1∶100 to determine the anti-PfMSP-1_19_ IgG antibody responses as previously described by Sachdeva et al. [53].

For western blot analyses, parasites were isolated from tightly synchronized cultures at different developmental stages by lyses of infected erythrocyte with 0.15% saponin. Parasite pellets were washed with PBS, suspended in Laemmli buffer, boiled, centrifuged, and the supernatant obtained was separated on 12% SDS–PAGE. The fractionated proteins were transferred from gel onto the nitrocellulose membrane (Amersham) and blocked in blocking buffer (1×PBS, 0.1% Tween-20, 5% milk powder) for 2 h. The blot was washed and incubated for 1 h with primary antibody [mouse anti-PfAARP (1∶500); rabbit anti-PfAARP (1∶1000); rabbit anti HRPII (1∶2000)] diluted in dilution buffer (1× PBS, 0.1% Tween-20, and 1% milk powder). Later, the blot was washed and incubated for 1 h with appropriate secondary antibody (anti-rabbit or anti-mouse, 1∶2000) conjugated to HRP, diluted in dilution buffer. Bands were visualized by using ECL detection kit (Amersham).

### COS cell expression plasmid constructs, COS cell culture, transfection and immunofluorescence assays

To generate COS cell expression plasmid constructs, two fragments of PfAARP one corresponding to complete gene except the signal sequence and the transmembrane region (F, 20aa-190aa), and other representing the N-terminal half (N, 20aa-92aa) were amplified from the genomic DNA using primers 554A (5′ CCG CAG CTG ATA TTG AGG AAT AAC AAA AGT C 3′) and 556A (5′ CGG GGG CCC TGG AGG TGG GGG AGG TAC 5′) for F fragment, and 554A and 555A (5′ CGG GGG CCC ATC TTC ATT GTC TTC TTC 3′) for N fragment. The amplified PCR products were digested with *Pvu*II and *Apa*I restriction enzymes and cloned into *Pvu*II and *Apa*I sites of pRE4 vector [10] in frame with the signal sequence and transmembrane segment of HSV gD. The resultant plasmids were labeled as pRE4-PfAARP-F and pRE4-PfAARP-N respectively.

The COS-7 cells were cultured in Dulbecco modified Eagle medium (DMEM; Invitrogen) with 10% heat-inactivated fetal calf serum (FCS) in a humidified CO_2_ (5%) incubator at 37°C. Fresh monolayers of 40% to 60% confluent COS-7 cells growing in 35-mm diameter wells were transfected with 2 to 4 µg plasmid DNA using Lipofectamine Plus reagent (Invitrogen) following manufacturer's instructions. To detect expression of the fusion proteins on the surface of transfected COS-7 cells and to calculate the transfection efficiency, immunofluorescence assays were performed , 36 to 40 hours after transfection, following Chitnis and Miller [11], using mouse monoclonal antibody DL6 (kindly provided by Drs Roselyn Eisenberg and Gary Cohen), which reacts with amino acids 272 to 279 of HSV gD.

### Erythrocyte binding assay with COS-7 cells expressing PfAARP on the surface

Human erythrocytes were washed three times in incomplete RPMI1640 media (Life Technologies, Inc.) and re-suspended in RPMI1640. Erythrocytes were treated with neuraminidase, chymotrypsin and trypsin as described earlier [11]. Briefly, for neuraminidase treatment 5.5 ml of a 5% (vol/vol) suspension of the washed human erythrocytes in incomplete medium was incubated twice with 3 milliunits of neuraminidase (*Vibrio cholerae*; Sigma) for 1 h at 37°C each time. For trypsin treatment, washed human erythrocytes were incubated with 1 mg/ml tosyl-phenylalanine-chloromethyl-ketone-treated trypsin (Sigma) for 1 h at 37°C. For chymotrypsin treatment, erythrocytes were incubated with 1 mg/ml α-chymotrypsin (Sigma) for 1 h at 37°C.

COS-7 cells transfected with constructs pRE4-PfAARP-F and pRE4-PfAARP-N were tested for binding to normal, neuraminidase-treated, chymotrypsin-treated and trypsin-treated human erythrocytes, 36 to 40 hours after transfection, following Chitnis and Miller [11]. COS-7 cells transfected with plasmid pHVDR22 [11] were used as positive control. The number of rosettes of erythrocytes bound to transfected COS-7 cells was scored in 20 fields at 200× magnification for each set. A cluster of 8 or more erythrocytes bound to a COS-7 cell was scored as a rosette. The number of rosettes observed was normalized for transfection efficiency of 5% for all the sets.

### Erythrocyte binding assay (EBA) with recombinant protein and antibody mediated inhibition of binding

Erythrocyte binding assays were carried out using recombinant PfAARP following Pandey et al [54]. About 100 µl of packed volume of normal human erythrocytes, previously washed with RPMI, were suspended in RPMI containing 10% FCS and 20 µg of recombinant protein to a final volume of 500 µl. This suspension was incubated for 1 h at room temperature with constant shaking. The reaction mixture was layered over dibutylpthalate (Sigma) and centrifuged to collect erythrocytes. Bound protein was eluted from the erythrocytes with 300 mM NaCl, separated by SDS-PAGE and detected by Western blotting using a commercially available mouse monoclonal antibody to penta-histidine (Qiagen). Recombinant *P. vivax* Duffy binding antigen region II (PvRII) and *P. falciparum* DNA helicase 60 (PfD60) were used as positive and negative controls respectively, in the RBC binding assay.

Efficacy of anti-PfAARP antibodies to inhibit the binding of PfAARP with human erythrocytes was also assessed in similar manner. Briefly, aliquots of recombinant protein (20 µg) were incubated at room temperature for 1 h with different amounts (25–100 µg) of IgG purified from the sera of rabbit immunized with PfAARP. After incubation these recombinant protein samples were used for erythrocyte binding assays. IgG purified from pre-immune sera were used as control.

### Fluorescence microscopy and indirect immunofluorescence assay

*P. falciparum* culture transfected with pTGFP-AARP was synchronized by two consecutive sorbitol treatments with 4 h gap between each treatment. Ahydrotetracycline was removed from the parasite cultures 72 h prior to live imaging (in the presence of 2.5 nM WR99210) to allow expression of the GFP fusion protein. Parasites at different developmental stages were collected for fluorescence microscopy and stained with DAPI at a final concentration of 2 µg/ml for 30 min at 37°C, prior to imaging. Fluorescence from DAPI and GFP was observed and captured from live cells using a Nikon TE 2000-U fluorescence microscope.

Indirect immunofluorescence assays were performed on *P. falciparum* 3D7 parasite lines as described earlier [55], [56]. Thin smears of *P. falciparum* infected erythrocytes were made on glass slide and fixed with a mixture of methanol/acetone. Slides were blocked in blocking buffer (1×PBS, 10% FCS) for 2 h at 37°C. After blocking, slides were incubated with primary antibody diluted in blocking buffer (mice anti-PfAARP, 1∶100; rabbit anti-AMA-1 1∶300; rabbit anti-EBA-175, 1∶300; rabbit anti-Clag3.1 1∶300; rabbit anti-MSP-1, 1∶500) for 1 h at 37°C. Slides were washed with 1× PBS for 1 h and incubated with appropriate secondary antibody conjugated to fluorescent dye (FITC or Cy3; dilution 1∶100) for 1 h. The slides were stained with DAPI for 30 min at 37°C at final concentration of 2 µg/ml and then washed twice in 1× PBS–Tween 0.05%, once in 1× PBS and mounted on a cover slip in the presence of anti fade mounting media (Bio-Rad). The slides were viewed on Nikon TE 2000-U fluorescence microscope.

### Cryo-Immuno-electron microscopy

Immuno-electron microscopy was carried out on *P. falciparum* 3D7 schizont stages parasites. Parasites were fixed in 4% paraformaldehyde, 0.04% glutaraldehyde in 1× PBS at 4°C for 1 h and subsequently embedded in gelatin, and infiltrated with a cryo preservative and plasticizer (2.3 M sucrose/20% polyvinyl pyrrolidone). After freezing in liquid nitrogen, samples are sectioned with a Leica Ultracut UCT cryo-ultramicrotome (Leica Microsystems Inc., Bannockburn, IL) at −60°C. Ultra thin sections were blocked with 5% fetal bovine serum and 5% normal goat serum in 1×PBS for 30 min and subsequently stained with anti-PfAARP antibody (1∶100 dilution in blocking buffer), washed thoroughly and incubated with 18 nm colloidal gold-conjugated mouse IgG (Jackson ImmunoResearch Laboratories, Inc., West Grove PA) for 1 hr. Sections were stained with 0.3% uranyl acetate/1.7% methyl cellulose and visualized under JEOL 1200EX transmission electron microscope (JEOL USA Inc., Peabody, MA). All labeling experiments were conducted in parallel with controls omitting the primary antibody or using pre-immune sera as primary antibodies.

### IgG purification

Total IgG was purified from the sera of rabbits immunized with PfAARP-N and also from rabbits immunized with adjuvant alone, following the procedure described previously [53]. Briefly, sera samples were loaded on a protein G-Sepharose column (Amarsham), equilibrated with the binding buffer (100 mM Tris-HCl, pH 8.0). The column was washed with 10-column volumes of the binding buffer. The bound IgG was eluted with 0.2 M glycine-HCl, pH 3.0 and the fractions were analyzed on SDS-PAGE. The fractions containing purified IgG were pooled and dialyzed against RPMI.

### Human sera samples

The sera samples were collected from individuals residing in a *P. falciparum* endemic area (Orissa, India) [57]. Sera samples were also collected from healthy individuals with no known history of malaria and who have never visited malaria transmission areas. The sera were collected with the consent of these individuals and approval of Human Volunteers Research Ethical Committee of ICGEB.

### Sequencing of PfAARP allele from different *P. falciparum* isolates

The entire PfAARP gene, excluding predicted signal sequence, was PCR amplified using high fidelity PCR enzyme mix (MBI Fermentas) and total genomic DNAs of five different *P. falciparum* strains and five field isolates (Table S1) using 500A/501A-primer pair. The resulting PCR products (∼600–650 bp) purified by the PCR purification kit (Qiagen) were used for direct sequencing. For each DNA sample, amplified products from two separate PCR were sequenced in both the forward and reverse direction using big dye termination chemistry. The sequence data were aligned using Seqman II and the ClustalW algorithm in MegAlign (DNAStar, Madison, WI).

### In vitro parasite growth inhibition assay

Growth inhibition assay was performed as previously described [53], [58]. Briefly, tightly synchronized *P. falciparum* 3D7 parasites at schizont stage were cultured in 96 well plates in which hematocrit and parasitemia were adjusted to 2.0% and 0.5% respectively. IgG purified from sera of rabbit immunized with PfAARP or adjuvant alone, were added to the parasite cultures at final concentrations of 0.5, 0.25, 0.125 and 0.0625 mg/ml. The cultures were incubated for 20 h to allow for schizont rupture and merozoite invasion. Each assay was performed in triplicate and the experiment was repeated twice. For microscopic analysis, smears were made from each well, stained with Giemsa, and the numbers of ring stage parasites per 5000 RBCs were determined and percentage ring stage parasitemia was calculated to assess the parasite invasion. Percentage inhibition of the parasite invasion was calculated using the following formula:
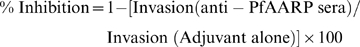



## Supporting Information

Figure S1 Expression and purification of N-terminal fragment of PfAARP. (A) SDS-PAGE showing purified recombinant N-terminal fragment of PfAARP, PfAARP-N, under reduced (lane 1) and non-reduced conditions (lane 2). (B) Reverse-phase HPLC profile of purified PfAARP-N showing a single homogenous population of recombinant protein that eluted as a single sharp peak.
(3.15 MB TIF)

Figure S2 Reactivity and specificity of anti-PfAARP rabbit antisera. (A) Western blot analysis of total parasite lysate (lane 1) and uninfected RBCs(lane 2) using anti-PfAARP-N antibodies detected a single specific band of ∼35 kDa in the parasites. (B) Western blot analysis of total parasite lysate using rabbit pre-immune sera. (C) Immuno-fluorescence assay showing reactivity of anti-PfAARP rabbit antibodies (red) with the schizont/merozoite stage parasites. The parasite nuclei were stained with DAPI (blue) and slides were visualized by fluorescence microscope. S, schizont and M, free merozoites. (D) Immunofluorescence assay showing co-immunostaining of *P. falciparum* transgenic parasites at schizont stages with anti-GFP (green)and anti-PfAARP rabbit (red) antibodies. The parasite nuclei were stained with DAPI (blue) and slides were visualized by fluorescence microscope.
(8.94 MB TIF)

Figure S3 Expression of PfAARP on COS cells surface and RBC binding assay. (A) Immunofluorescence assay of COS cells trasfected with pRE4-PfAARP construct, using anti-PfAARP antibodies. (B) RBC binding assay of transfected COS cells using human erythrocytes.
(3.20 MB TIF)

Figure S4 Scatter plots representing ELISA results using sera from individuals residing in *P. falciparum* endemic areas; each serum was tested in triplicate against recombinant PfAARP-N (A), recombinant PfMSP-1_19_ (B) was kept as positive control. The horizontal bars indicate the cutoff value (mean +2SD of negative controls) of the reactivity for positive responders. Sera samples from healthy individuals with no past history of malaria and who have never visited malaria transmission areas were used as controls.
(5.40 MB TIF)

Figure S5 Amino acid sequence alignment of PfAARP gene sequenced from five *P. falicparum* laboratory strains and five field isolates. Amino acids that are identical in at least six of the ten sequences (>60%) are shown in grey.
(8.44 MB TIF)

Table S1 Table showing details of *P. falciparum* strains and field isolates used for sequencing of PfAARP genes
(0.04 MB DOC)
