# Supplementary figures and images for: Identification and Characterization of a Novel Plasmodium falciparum Merozoite Apical Protein Involved in Erythrocyte Binding and Invasion
Source: PLoS One. 2008 Mar 5;3(3):e1732. doi: 10.1371/journal.pone.0001732 (PMC2253826; doi:10.1371/journal.pone.0001732)

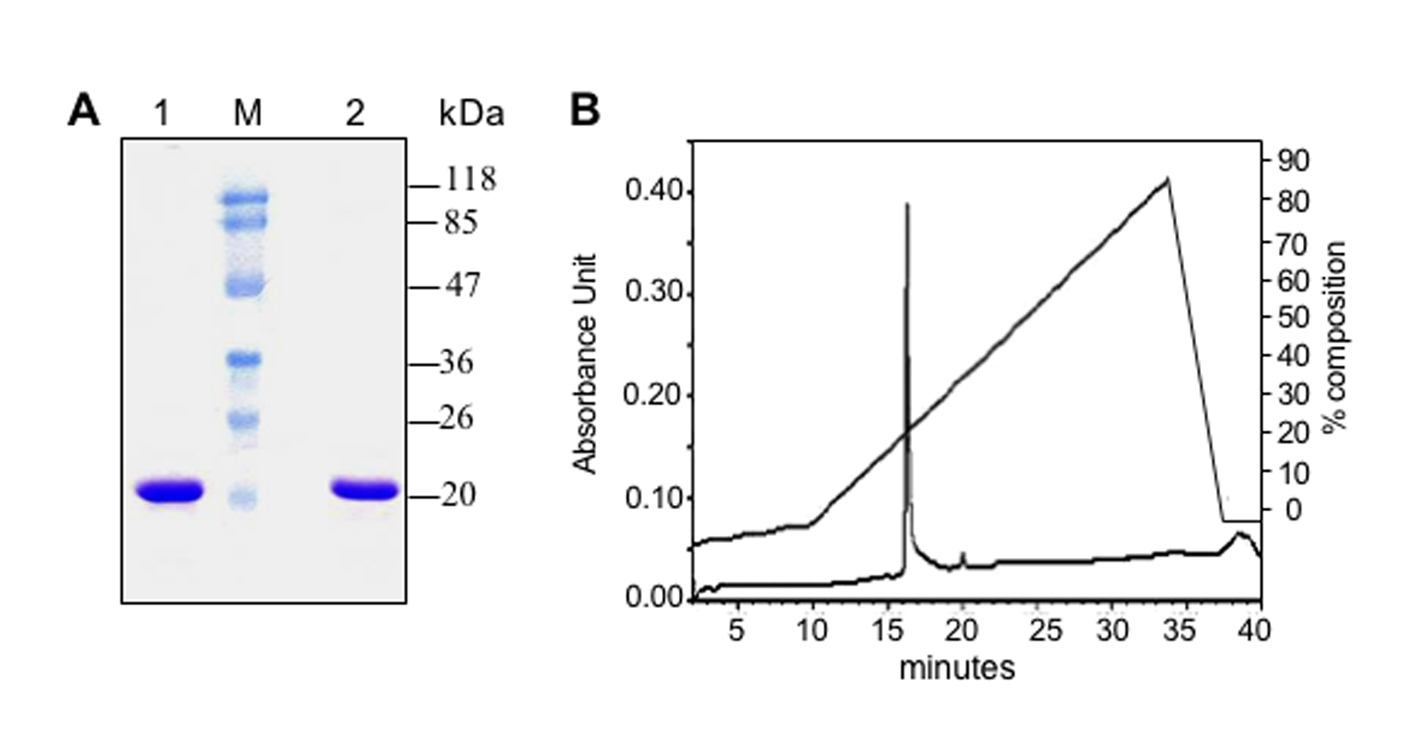

Supplement: Figure S1 — Expression and purification of N-terminal fragment of PfAARP. (A) SDS-PAGE showing purified recombinant N-terminal fragment of PfAARP, PfAARP-N, under reduced (lane 1) and non-reduced conditions (lane 2). (B) Reverse-phase HPLC profile of purified PfAARP-N showing a single homogenous population of recombinant protein that eluted as a single sharp peak. (3.15 MB TIF) [file pone.0001732.s001.tif]

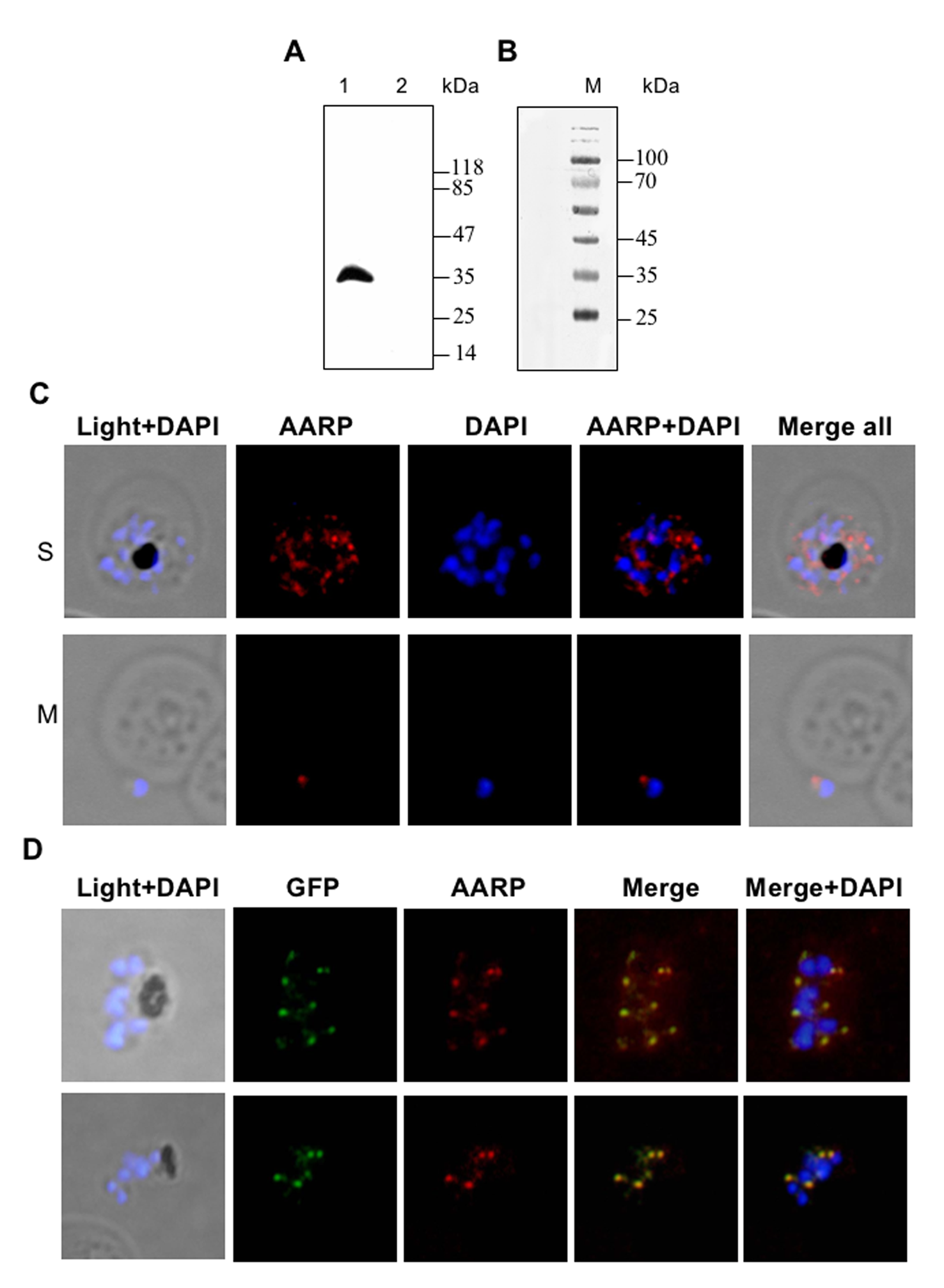

Supplement: Figure S2 — Reactivity and specificity of anti-PfAARP rabbit antisera. (A) Western blot analysis of total parasite lysate (lane 1) and uninfected RBCs(lane 2) using anti-PfAARP-N antibodies detected a single specific band of ∼35 kDa in the parasites. (B) Western blot analysis of total parasite lysate using rabbit pre-immune sera. (C) Immuno-fluorescence assay showing reactivity of anti-PfAARP rabbit antibodies (red) with the schizont/merozoite stage parasites. The parasite nuclei were stained with DAPI (blue) and slides were visualized by fluorescence microscope. S, schizont and M, free merozoites. (D) Immunofluorescence assay showing co-immunostaining of P. falciparum transgenic parasites at schizont stages with anti-GFP (green)and anti-PfAARP rabbit (red) antibodies. The parasite nuclei were stained with DAPI (blue) and slides were visualized by fluorescence microscope. (8.94 MB TIF) [file pone.0001732.s002.tif]

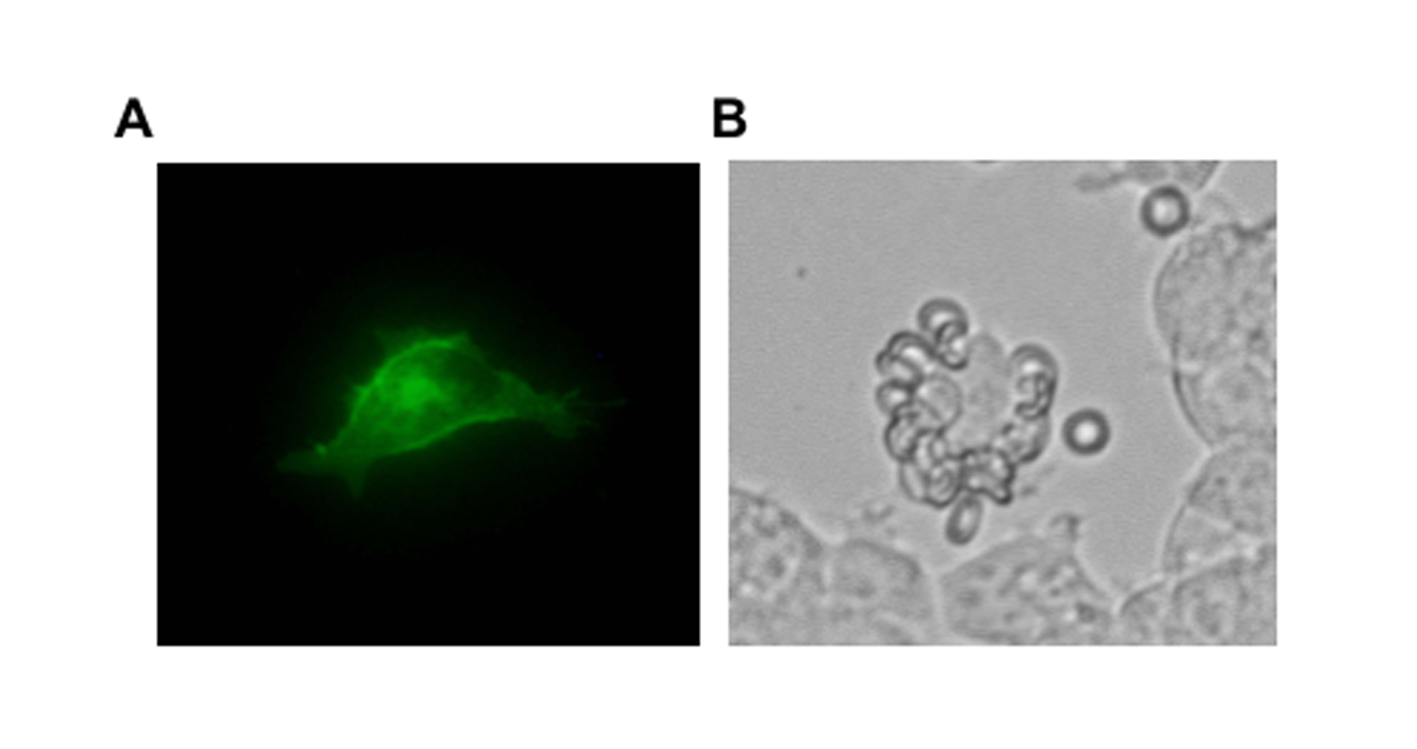

Supplement: Figure S3 — Expression of PfAARP on COS cells surface and RBC binding assay. (A) Immunofluorescence assay of COS cells trasfected with pRE4-PfAARP construct, using anti-PfAARP antibodies. (B) RBC binding assay of transfected COS cells using human erythrocytes. (3.20 MB TIF) [file pone.0001732.s003.tif]

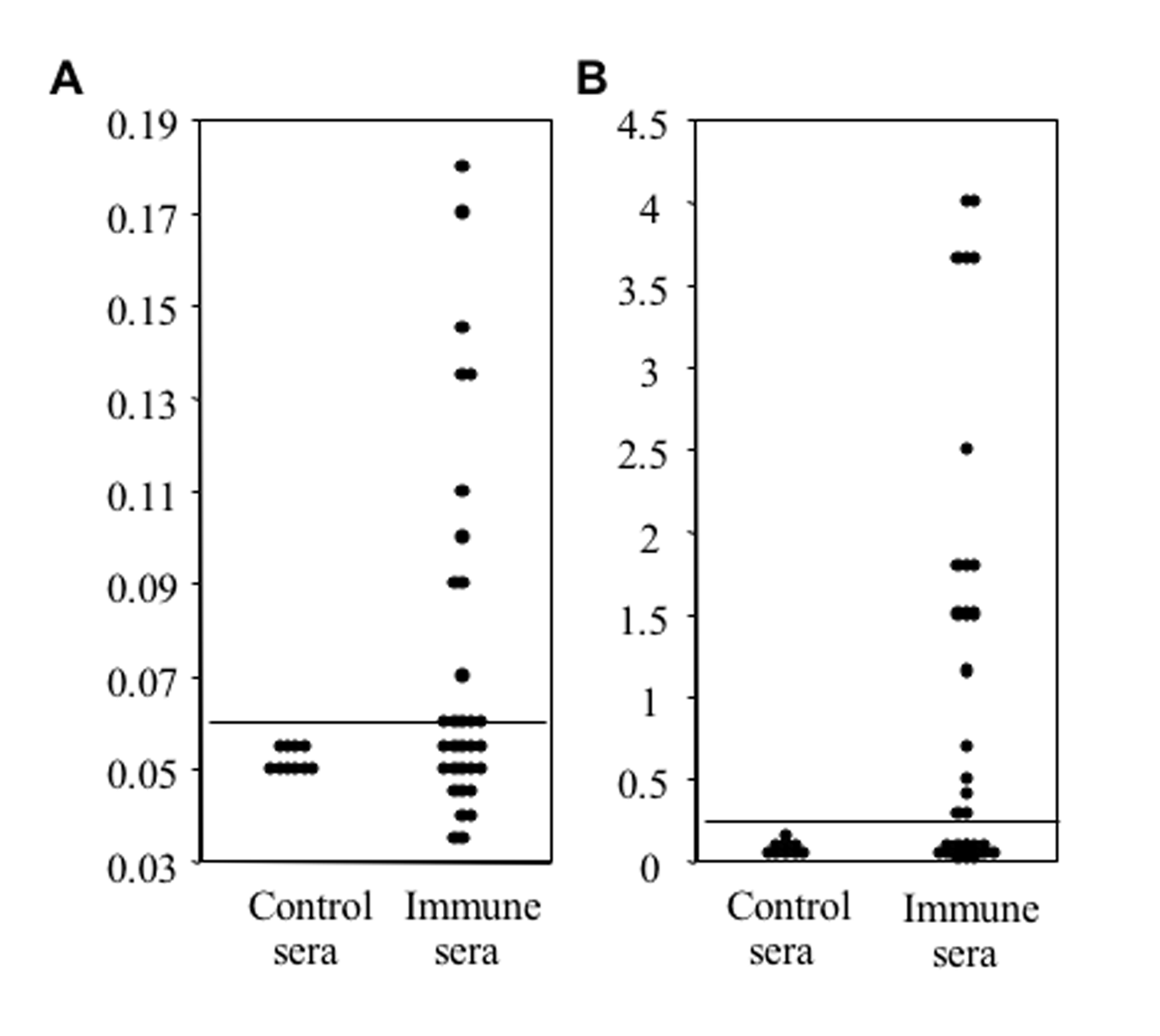

Supplement: Figure S4 — Scatter plots representing ELISA results using sera from individuals residing in P. falciparum endemic areas; each serum was tested in triplicate against recombinant PfAARP-N (A), recombinant PfMSP-119 (B) was kept as positive control. The horizontal bars indicate the cutoff value (mean +2SD of negative controls) of the reactivity for positive responders. Sera samples from healthy individuals with no past history of malaria and who have never visited malaria transmission areas were used as controls. (5.40 MB TIF) [file pone.0001732.s004.tif]

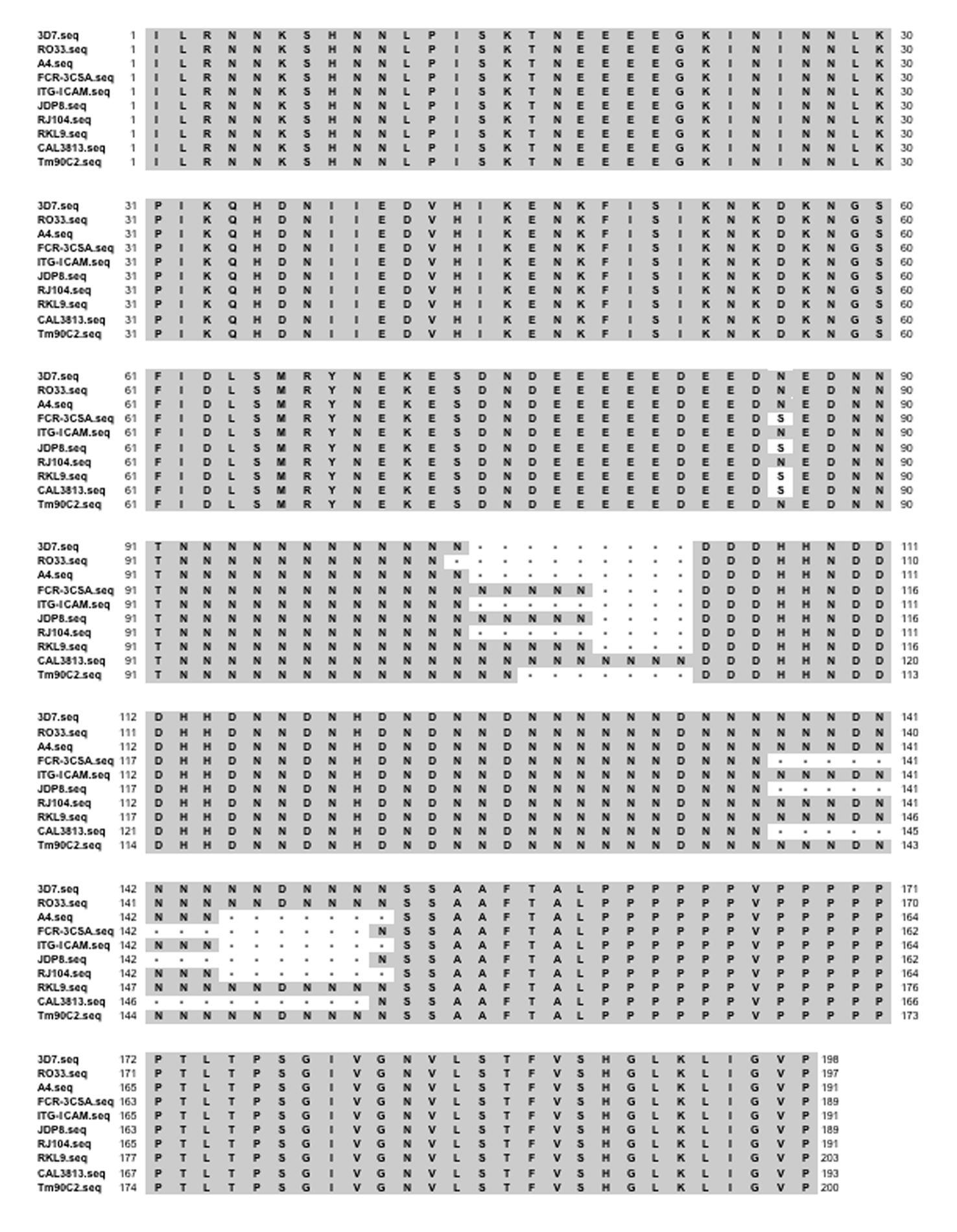

Supplement: Figure S5 — Amino acid sequence alignment of PfAARP gene sequenced from five P. falicparum laboratory strains and five field isolates. Amino acids that are identical in at least six of the ten sequences (>60%) are shown in grey. (8.44 MB TIF) [file pone.0001732.s005.tif]
