# Supplementary material for: Identification and Characterization of a Novel Plasmodium falciparum Merozoite Apical Protein Involved in Erythrocyte Binding and Invasion
Source: PLoS One. 2008 Mar 5;3(3):e1732. doi: 10.1371/journal.pone.0001732 (PMC2253826; doi:10.1371/journal.pone.0001732)
Supplement: Table S1 — Table showing details of P. falciparum strains and field isolates used for sequencing of PfAARP genes (0.04 MB DOC) [file pone.0001732.s006.doc]

**Table S1:** Table showing details of *P. falciparum* strains and field isolates used for sequencing of PfAARP genes

| Parasite isolate^1^ |  | Collection site |  | Source/Reference |
| --- | --- | --- | --- | --- |
|  |  |  |  |  |
| 3D7 |  | - |  | MR4 |
| A4 |  | - |  | [59] |
| ITG-ICAM |  | - |  | MR4 |
| FCR-3 CSA |  | - |  | MR4 |
| RO33 |  | - |  | MR4 |
| RKL-9 |  | Orissa |  | [60] |
| JDP8 |  | Madhya Pradesh |  | [60] |
| RAJ104 |  | Rajasthan |  | [60] |
| CAL-3813 |  | Calcutta |  | Chitnis, C. unpublished data |
| Tm90C2A |  | Thailand |  | Chitnis, C. unpublished data |
|  |  |  |  |  |

^1^Genotyping of each field isolate was confirmed by PCR amplification of *P. falciparum* Merozoite Surface Protein-1 and 2 (MSP-1 & MSP-2) genes [60 and Chitnis, C. unpublished data].
